# Greater Progression of Age-Related Aortic Stiffening in Adults with Poor Trunk Flexibility: A 5-Year Longitudinal Study

**DOI:** 10.3389/fphys.2017.00454

**Published:** 2017-06-30

**Authors:** Yuko Gando, Haruka Murakami, Kenta Yamamoto, Ryoko Kawakami, Harumi Ohno, Susumu S. Sawada, Nobuyuki Miyatake, Motohiko Miyachi

**Affiliations:** ^1^Department of Physical Activity Research, National Institute of Health and Nutrition, NIBIOHNTokyo, Japan; ^2^Faculty of Pharmaceutical Sciences, Teikyo Heisei UniversityTokyo, Japan; ^3^Faculty of Sport Sciences, Waseda UniversitySaitama, Japan; ^4^Department of Hygiene, Faculty of Medicine, Kagawa UniversityKagawa, Japan

**Keywords:** arteriosclerosis, aging, fitness, trunk flexibility, primary prevention

## Abstract

**Purpose:** Having a low level of physical fitness, especially cardiorespiratory fitness, appears to accelerate age-related aortic stiffening. Whereas, some studies have reported that trunk flexibility is a component of physical fitness, it is also negatively associated with arterial stiffening independent of cardiorespiratory fitness in cross-sectional studies. However, no long-term longitudinal study has determined whether poor trunk flexibility accelerates the progression of age-related aortic stiffening. We examined trunk flexibility and aortic stiffness progression in a 5-year longitudinal study.

**Methods and Results:** A total of 305 apparently healthy men and women participated in this study (49.6 ± 9.5 years of age). Trunk flexibility was measured using a sit-and-reach test. Aortic stiffness was assessed using carotid-femoral pulse wave velocity (cfPWV) at baseline and after 5 years. Analysis of covariance (ANCOVA) was used to assess the association of the annual rate of cfPWV across flexibility levels (low, middle, high). There were no significant differences in baseline cfPWV among the three groups (835 ± 164, 853 ± 140, 855 ± 2.68 cm/s; *P* = 0.577). Annual ΔcfPWV was significantly higher in the low-flexibility group than in the high-flexibility group (*P* = 0.009). ANCOVA revealed an inverse relationship between flexibility level and annual ΔcfPWV (14.41 ± 2.73, 9.79 ± 2.59, 2.62 ± 2.68 cm/s/year; *P* for trend = 0.011). Multiple regression analysis revealed that baseline sit and reach (β = −0.12, −0.70 to −0.01) was independently correlated with ΔcfPWV following adjustment for baseline peak oxygen uptake, age, sex, body fat, heart rate, and cfPWV. The 5-year change in cfPWV was not significantly correlated with 5-year change in sit-and-reach performance (*P* = 0.859).

**Conclusion:** Poor trunk flexibility is associated with greater progression of age-related aortic stiffening in healthy adults. However, we failed to confirm a significant association between 5-year change in aortic stiffness and 5-year change in trunk flexibility. The association between increased age-related increase in aortic stiffness and deterioration in flexibility due to age may require observation for more than 5 years.

## Introduction

Aortic stiffness, as indexed based on pulse wave velocity (PWV), increases progressively with advancing age (Lakatta, 2003) and is a major risk factor for cardiovascular disease and all-cause mortality (Vlachopoulos et al., 2010).

Having a high level of physical fitness, especially higher cardiorespiratory fitness, is associated with the suppression of age-related arterial stiffening (Vaitkevicius et al., 1993; Gando et al., 2016). Recently, we and others have reported that flexibility, a component of physical fitness (Cureton, 1941), is also negatively associated with arterial stiffening independent of cardiorespiratory fitness in cross-sectional studies (Yamamoto et al., 2009; Nishiwaki et al., 2014). It was reported that healthy children with stiffer skin and joints have higher blood pressure and higher pulse pressure levels, independent of several confounders (Uiterwaal et al., 2003). Moreover, the loss of trunk flexibility with aging accelerates at the fourth or fifth decade of life, based on 6,000 Flexitest results (Medeiros et al., 2013). The same tendency is observed for age-related arterial stiffening. However, no long-term longitudinal study has determined whether poor trunk flexibility accelerates the progression of age-related arterial stiffening.

Flexibility (body stiffness), which can be measured non-invasively, easily, quickly, and safely, may represent the phenotypic stiffness of various body parts. Flexibility is determined by connective tissue in the tendons, muscles, ligaments, and joint capsules (Alter, 2004). Increased aortic PWV is linked to structural alterations in the arterial wall, including increased connective tissue (Lakatta, 2003). Some studies have reported that benign joint hypermobility syndrome and Ehlers-Danlos syndrome, one of the most common heritable disorders of connective tissue, are characterized by joint laxity and associated with increased aortic compliance (Neil-Dwyer et al., 1983; Handler et al., 1985), distensibility (Boutouyrie et al., 2004), and lower aortic stiffness (Francois et al., 1986; Yazici et al., 2004). It is possible that poor flexibility may accelerate the progression of age-related arterial stiffening. Therefore, we hypothesized that the age-related increases in aortic stiffness were higher in individuals with low flexibility compared to those with high flexibility. The aim of this study was to investigate the association between flexibility and the progression of aortic stiffening in a longitudinal study.

## Methods

### Participants

The study population is part of the Nutrition and Exercise Intervention Study (NEXIS) cohort, registered at Clinical Trials.gov, identifier: NCT00926744. For the purpose of this study, a total of 305 Japanese adults (80 men and 225 women; mean age, 49.6 ± 9.5 years; range, 27–66 years) were selected from among 1,125 participants. Participants underwent anthropometric measurements, physical fitness testing (cardiorespiratory fitness and flexibility), physical activity assessments, arterial stiffness measurement, and blood examinations (baseline measurement). They underwent the same tests (except for physical activity) at 5-year follow-up (follow-up measurement). We excluded participants with a history of stroke, cardiac disease, or chronic renal failure, and those undergoing medical treatment for hypertension, dyslipidemia, or diabetes. We also excluded current smokers. Participants in this sample had an ankle-brachial pressure index between 0.9 and 1.3 at both baseline and the follow-up visit (during the observation period). All participants gave their written informed consent before participating in the study. The study was approved by the ethical committees of the National Institutes of Biomedical Innovation, Health and Nutrition, and Okayama Health Foundation, and the study was performed in accordance with the guidelines of the Declaration of Helsinki.

### Arterial stiffness and blood pressure

We measured carotid and femoral PWV (cfPWV) as indicators of aortic stiffness and blood pressure with a vascular test device (form PWV/ABI; Omron Colin, Japan) as described previously (Gando et al., 2016). Waveforms were measured by applanation tonometry according to a standardized protocol. The standard deviation of the difference for interobserver reproducibility was 62 cm/s in our laboratory (Gando et al., 2010). Heart rate (HR) was simultaneously determined during the measurement of cfPWV (form PWV/ABI; Omron Colin, Japan). Recordings were made in triplicate, with participants in the supine position, and conformed strictly to American Heart Association guidelines (Pickering et al., 2005). The mean right and left brachial BPs were used for analysis.

### Flexibility

We measured sit-and-reach test performance as an indicator of trunk flexibility with a trunk flexion meter (T.K.K.5112; Takeikiki, Japan) as described previously (Yamamoto et al., 2009). Participants sat on the floor with legs stretched out straight in front of the body. They put both hands on the trunk flexion meter and flexed forward slowly. The device then displayed the distance moved. The standard deviation of the difference for interobserver reproducibility was 2.3 cm in our laboratory (Yamamoto et al., 2009).

Participants were classified into low, moderate, or high trunk flexibility categories according to the distribution of sex- and age (20–29, 30–39, 40–49, 50–59, and 60–69 years) -specific sit and reach test results: lowest tertile, poor flexibility; middle tertile, mid-range flexibility; and highest tertile, high flexibility.

### Cardiorespiratory fitness

We measured peak oxygen uptake as an indicator of cardiorespiratory fitness, which was measured according to the protocol of a graded exercise load using a cycle ergometer (Ergomedic 828E Test Cycle, Monark, Sweden, or Excalibur V2.0, The Netherlands), as described previously (Gando et al., 2016).

### Physical activity

We assessed physical activity using triaxial accelerometry (Actimarker EW4800; Panasonic Electric Works, Japan), as described previously (Gando et al., 2010). Participants wore a triaxial accelerometer for 28 days with habitual physical activity. They were instructed to wear the accelerometer from the time they woke up until they went to bed. We used the data for at least 14 days (2 weeks) and obtained daily physical activity durations corresponding to 1.1 to 2.9 metabolic equivalents (METs) (light), 3.0 to 5.9 METs (moderate), or ≥6.0 METs (vigorous) (Haskell et al., 2007).

### Body composition

We assessed body composition using dual-energy X-ray absorptiometry (Hologic QDR-4500; Hologic, Waltham, MA). We measured waist circumference using a tape measure. Body mass index (BMI) was calculated as measured weight in kilograms divided by the square of measured height in meters.

### Blood samples

Blood samples were obtained after at least 10 h of overnight fasting. Fasting plasma glucose and glycated hemoglobin (HbA1c) and serum levels of total cholesterol, high-density lipoprotein (HDL) cholesterol, and triglycerides were measured.

### Statistical analyses

Means ± standard deviations were calculated for continuous variables. Analysis of variance (ANOVA) was used to assess the relationship of the continuous variables to categories of flexibility levels. The differences between baseline and follow-up measurements were assessed by paired *t*-test and McNemar's non-parametric test. In the ANOVA, Scheffe's method was used to identify significant differences among mean values.

Pearson's correlation coefficients were used to analyze the relationships between the 5-year changes in cfPWV and baseline values of factors known to influence vascular stiffness and 5-year changes in these variables.

Analysis of covariance (ANCOVA) models were estimated to test differences in the annual rate of cfPWV [annual ΔcfPWV: (follow-up cfPWV—baseline cfPWV)/follow-up years] across flexibility levels. The annual ΔcfPWV was entered as a dependent variable; the tertile flexibility category was entered as a fixed factor; and baseline age, weight, body fat, systolic blood pressure (SBP), HR, cfPWV, peak oxygen uptake, moderate physical activity time, vigorous physical activity time, and sex were entered as covariates for adjustment. Pairwise *post-hoc* comparisons were examined using a Bonferroni test. In ANCOVA, data were expressed as estimated marginal mean ± standard error.

A multiple regression analysis was used to determine the influences of baseline values of factors known to influence vascular stiffness and changes in these variables (annual rate of change) on the annual ΔcfPWV.

*P* values < 0.05 were considered statistically significant. Data were analyzed using SPSS software (IBM Japan v.20.0, Japan).

## Results

Table 1 shows the baseline characteristics and changes in these variables of the participants divided by flexibility levels. There were no significant differences in baseline cfPWV among the three groups (*P* = 0.577). The paired *t*-test demonstrated that cfPWV increased significantly during the follow-up period in low and middle flexibility groups. The body fat, HbA1c, SBP increased significantly during the follow-up period in all flexibility groups.

**Table 1:** Changes in participants' characteristics during the study period.

**Variables**	**Low**		**Middle**		**High**	
	**Baseline**	**Follow-up**	**Baseline**	**Follow-up**	**Baseline**	**Follow-up**
*N* (men/women)	99 (23/76)		104 (30/74)		102 (27/75)	
Follow-up year, years	5.0 ± 0.1		5.0 ± 0.1		5.0 ± 0.1	
Sit-and-reach, cm	30.2 ± 6.0	31.1 ± 7.8	40.0 ± 4.8	38.7 ± 7.7^†^	49.7 ± 5.5	48.7 ± 7.1^†^
Premenopausal Women, *n* (%)	31 (31)	19 (19)^†^	30 (29)	23 (22)	34 (33)	22 (22)^†^
Age, years	49.8 ± 9.4	54.8 ± 9.4^†^	49.4 ± 9.7	54.4 ± 9.7^†^	49.5 ± 9.5	54.6 ± 9.5^†^
Height, cm	159.7 ± 8.3	159.4 ± 8.3^†^	160.7 ± 7.5	160.4 ± 7.5^†^	162.6 ± 8.6^*^	162.2 ± 8.6^*^^†^
Weight, kg	57.3 ± 9.7	56.9 ± 9.4	58.2 ± 9.1	58.4 ± 8.9	58.1 ± 9.2	58.1 ± 9.6
BMI, kg/m^2^	22.3 ± 2.8	22.3 ± 2.8	22.4 ± 2.4	22.5 ± 2.3	21.9 ± 2.5	22.0 ± 2.8
Waist circumference, cm	81.0 ± 8.3	81.1 ± 7.7	80.2 ± 7.6	81.6 ± 7.6^†^	79.1 ± 8.4	79.3 ± 8.5
Body fat, %	27.4 ± 6.2	28.0 ± 6.6^†^	25.8 ± 6.3	27.0 ± 6.4^†^	24.1 ± 7.0^*^	25.9 ± 7.4^†^
Glucose, mg/dL	89.3 ± 8.1	86.7 ± 9.9^†^	90.6 ± 13.1	88.6 ± 19.3^†^	89.7 ± 10.3	85.4 ± 9.9^†^
HbA1c, %	5.3 ± 0.3	5.4 ± 0.3^†^	5.3 ± 0.5	5.5 ± 0.7^†^	5.3 ± 0.4	5.4 ± 0.3^†^
Total cholesterol, mg/dL	212 ± 38	221 ± 36^†^	213 ± 33	219 ± 35	208 ± 36	217 ± 35^†^
HDL cholesterol, mg/dL	67 ± 20	71 ± 20^†^	65 ± 17	67 ± 20	68 ± 16	71 ± 17^†^
Triglycerides, mg/dL	88 ± 47	103 ± 182	97 ± 71	100 ± 69	81 ± 54	77 ± 38
SBP, mmHg	117 ± 14	120 ± 16^†^	115 ± 12	117 ± 13^†^	118 ± 13	121 ± 15^†^
DBP, mmHg	71 ± 11	73 ± 11^†^	70 ± 9	71 ± 10^†^	71 ± 10	72 ± 10
HR, beats per minute	62 ± 10	61 ± 9	62 ± 11	61 ± 9	62 ± 12	60 ± 10
cfPWV, cm/s	835 ± 164	913 ± 212^†^	853 ± 140	895 ± 167^†^	855 ± 140	871 ± 156
*N* (men/women)	99 (23/76)	94 (23/71)	104 (30/74)	95 (28/67)	102 (27/75)	94 (26/68)
Peak oxygen uptake, mL/kg/min	29.7 ± 5.9	30.0 ± 7.2	31.8 ± 7.8	31.5 ± 7.8	33.1 ± 7.3^*^	34.0 ± 9.0^*^
*N* (men/women)	99 (23/76)	–	104 (30/74)	–	102 (27/75)	–
Daily time spent in physical activity						
Light, min/day	586 ± 117	–	571 ± 115	–	587 ± 106	–
Moderate, min/day	55 ± 21	–	63 ± 25	–	64 ± 4^*^	–
Vigorous, min/day	1.7 ± 6.3	–	2.8 ± 5.9	–	3.8 ± 7.7	–

*Values are mean ± SD, or n (%)*.

* *P < 0.05 vs. Low (assessed by 1-way analysis of variance with post-hoc multiple comparisons by Scheffe's test)*.

† *P < 0.05 vs. Baseline (assessed by paired t-test for continuous variables, and by McNemar's non-parametric test for premenopausal women)*.

*BMI indicates body mass index; HbA1c, hemoglobin A1c; HDL cholesterol, high-density lipoprotein cholesterol; SBP, systolic blood pressure; DBP, diastolic blood pressure; and cfPWV, carotid-femoral pulse wave velocity*.

Pearson's correlation coefficients between the 5-year change in cfPWV and baseline and 5-year changes in other parameters were as follows: the 5-year change in cfPWV was correlated with baseline sit-and-reach (*r* = −0.16, *P* = 0.005); weight (*r* = 0.15, *P* = 0.007); BMI (*r* = 0.15, *P* = 0.011); waist (*r* = 0.13, *P* = 0.029); cfPWV (*r* = −0.26, *P* < 0.001); peak oxygen uptake (*r* = −0.12, *P* = 0.030); and 5-year changes in SBP (*r* = 0.24, *P* < 0.001), diastolic blood pressure (DBP) (*r* = 0.24, *P* < 0.001), and HR (*r* = 0.21, *P* < 0.001). The 5-year change in cfPWV was not significantly correlated with the 5-year-change in sit-and-reach. Moreover, univariate regression analyses were used to assess the relationships between sit-and-reach values and the 5-year changes of cfPWV in different age categories (young, middle, old). The Pearson's correlation coefficient was larger in older subjects than in young and middle-aged subjects (young, *r* = −0.13; middle-aged, *r* = −0.15; older, *r* = −0.26). These results suggest that poor trunk flexibility is associated with greater progression of age-related aortic stiffening, especially in older adults.

**Figure 1:**
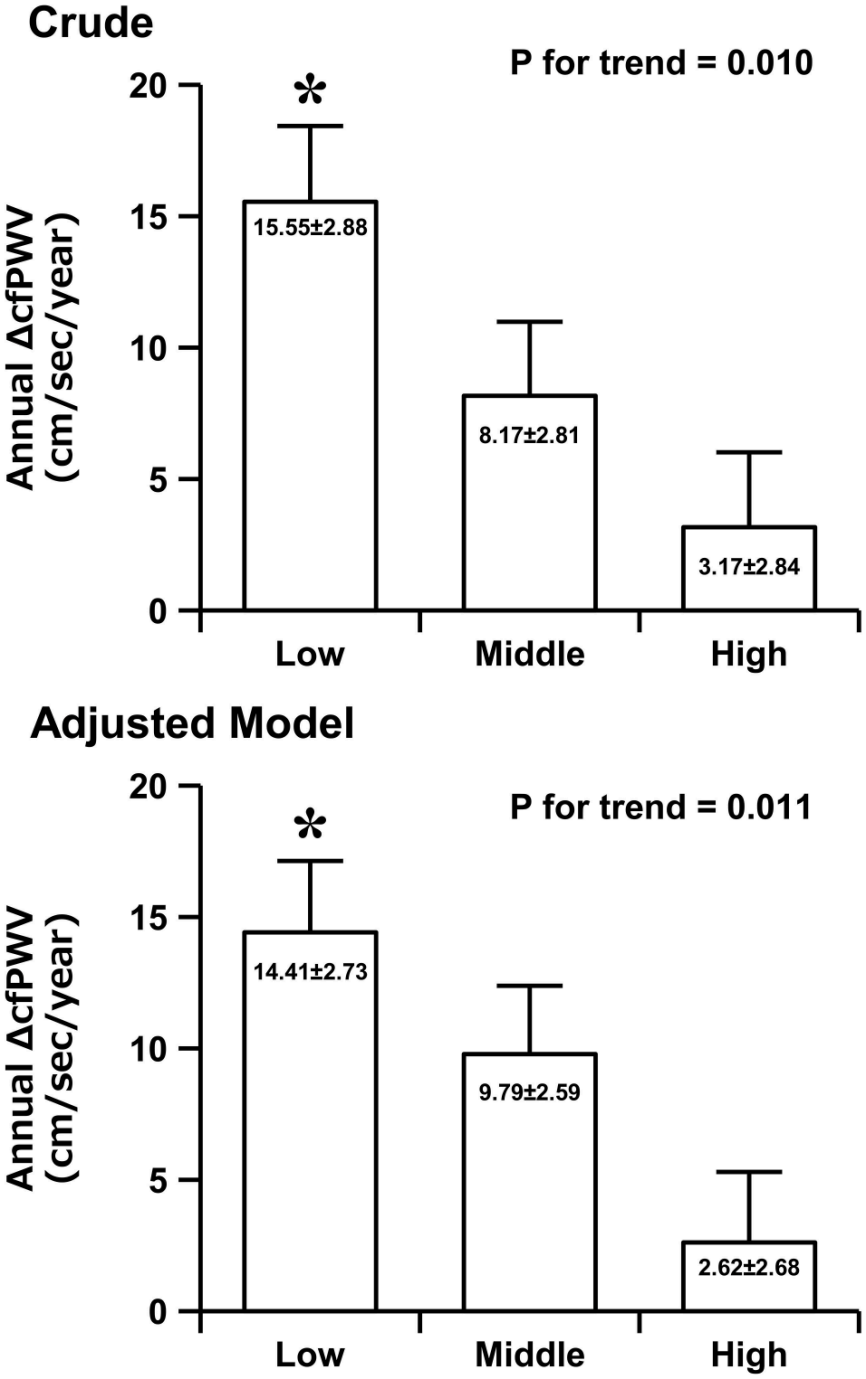
The crude and adjusted values of the annual rate of change in carotid-femoral pulse wave velocity (ΔcfPWV) across the flexibility levels. Data (adjusted model) are adjusted for baseline age, weight, body fat, SBP, HR, cfPWV, peak oxygen uptake, moderate physical activity time, vigorous physical activity time, and sex. Data are presented as mean ± standard error. ^*^P < 0.05 vs. the high-flexibility group.

Figure 1 shows the crude and adjusted values of the annual ΔcfPWV across flexibility levels. ANCOVA revealed an inverse relationship between flexibility level and the annual ΔcfPWV (14.41 ± 2.73, 9.79 ± 2.59, 2.62 ± 2.68 cm/s/year; *P* for trend = 0.011). The annual ΔcfPWV was significantly higher in the low-flexibility group than in the high-flexibility group (*P* = 0.009). Moreover, we performed ANCOVA analyses by sex. In men, ANCOVA revealed an inverse relationship between flexibility level and the annual ΔcfPWV (21.86 ± 6.65, 15.40 ± 5.46, −0.94 ± 6.00 cm/s/year; *P* for trend = 0.044). In women, ANCOVA revealed an inverse relationship (not statistically significant) between flexibility level and annual ΔcfPWV (11.23 ± 3.00, 7.68 ± 2.95, 4.67 ± 3.01 cm/s/year; *P* for trend = 0.329).

As shown in Table 2, multiple regression analysis revealed that baseline sit-and-reach and peak oxygen uptake were independent correlates of annual ΔcfPWV (Model 1). Upon further adjustment including body fat, HR, and cfPWV (Model 2), baseline sit-and-reach and peak oxygen uptake were independent correlates of annual ΔcfPWV. Annual ΔHR was independently correlated with annual ΔcfPWV (Model 3). However, annual Δsit-and-reach, Δpeak oxygen uptake, and Δbody fat were not independent correlates of annual ΔcfPWV.

**Table 2:** Multiple regression analysis showing the influences of baseline and changes in these variables (annual rate of change) on the annual ΔcfPWV.

	**Non-standardized coefficient**	**95% CI**		**Standardized coefficient**	***P*-value**
Δ**cfPWV**	**Model 1 (*****R**^2^* **changes** = **0.047)**				
Sit-and-reach, cm	−0.37	−0.73	−0.01	−0.12	0.043
Peak oxygen uptake, mL/kg/min	−0.72	−1.3	−0.13	−0.18	0.016
Age, years	−0.12	−0.52	0.27	0.04	0.537
Sex, men = 0; women = 1	−6.73	−15.51	2.06	−0.10	0.133
Δ**cfPWV**	**Model 2 (*****R**^2^* **changes** = **0.151)**				
Sit-and-reach, cm	−0.36	−0.70	−0.01	−0.12	0.043
Peak oxygen uptake, mL/kg/min	−0.86	−1.52	−0.22	−0.21	0.010
Age, years	0.41	−0.02	0.84	0.13	0.063
Sex, men = 0; women = 1	−12.0	−21.42	−2.50	−0.18	0.013
Body fat, %	−0.35	−1.10	0.40	−0.08	0.361
HR, beats per minute	0.02	−0.29	0.34	0.01	0.877
cfPWV, cm/s	−0.07	−0.10	−0.05	−0.36	0.000
Δ**cfPWV**	**Model 3 (*****R***^2^ **changes** = **0.083)**				
ΔSit-and-reach, cm	−0.33	−3.96	3.31	−0.01	0.859
ΔPeak oxygen uptake, mL/kg/min	−2.14	−6.93	2.65	−0.07	0.380
ΔBody fat, %	−4.03	−10.67	2.62	−0.10	0.233
ΔHR, beats per minute	3.61	1.42	5.81	0.26	0.001

*cfPWV indicates carotid-femoral pulse wave velocity; 95% CI, 95% confidence interval*.

## Discussion

In this 5-year longitudinal study, the age-related increases in aortic stiffness were higher in the low-flexibility groups compared with those in the high-flexibility groups. Moreover, trunk flexibility was inversely associated with the annual rate of change in cfPWV. This association was independent of other confounders. These findings suggest that poor trunk flexibility is associated with greater progression of age-related aortic stiffening in healthy adults.

The current study has several strengths that set it apart from previous studies. Previous cross-sectional studies indicated that poor trunk flexibility was associated with arterial stiffening (Yamamoto et al., 2009; Nishiwaki et al., 2014). However, to our knowledge, there have been no long-term longitudinal studies with repeated measures of cfPWV and assessment of other risk factors regarding the relationships between flexibility and the progression of aortic stiffness. Here, we determined the relationship between flexibility and the 5-year change in cfPWV. It is well-known that cardiorespiratory fitness is inversely related to arterial stiffness (Vaitkevicius et al., 1993; Gando et al., 2016). Similar to previous findings, the present study also confirmed that arterial stiffness was significantly related to cardiorespiratory fitness. More importantly, the present study demonstrated that trunk flexibility, which has been identified as a component of physical fitness (Cureton, 1941), was also inversely related with arterial stiffening, independent of cardiorespiratory fitness and other confounders. The present findings statistically support the hypothesis that flexibility is a predictor of arterial stiffness, independent of cardiorespiratory fitness and other confounders. By contrast, the 5-year change in cfPWV was not significantly correlated with the 5-year-change in sit-and-reach performance. This result was unexpected, because some studies have suggested that stretching (Nishiwaki et al., 2015) and yoga (Hunter et al., 2013) exercise interventions significantly improved sit-and-reach performance and arterial compliance and reduced arterial stiffness. Our data indicate that the 5-year change in sit-and-reach was small (mean 5-year change, −0.45 ± 5.44 cm), which may be hardly detectable. A significant correlation between the changes in cfPWV and sit-and-reach might be detectable over a longer-term follow-up period. Further research is needed to understand this association.

On the other hand, similar to a previous study (Tomiyama et al., 2010), the present study also showed that the 5-year changes in HR was independently correlated with 5-year change in cfPWV. Moreover, flexibility is influenced by age and environmental and genetic factors. Age is associated with both flexibility (Medeiros et al., 2013) and arterial stiffness (Lakatta, 2003). Environmental factors such as exercise habits or physical activity levels are also associated with flexibility (Harvey et al., 2002) and arterial stiffness (Gando et al., 2010). Our findings demonstrate that flexibility is associated greater progression of age-related aortic stiffening, independent of age or physical activity level. Thus, flexibility might be an independent factor of age-related arterial stiffening. Our recent study indicated that ACTN3 R577X genotype could be an independent genetic factor of trunk flexibility (Kikuchi et al., 2017). This study indicated that RR genotype was associated with significantly lower flexibility than XX. Moreover, Deschamps et al. (2015) reported that RR genotype was associated with higher systolic and diastolic blood pressure. Blood pressure is a strong determinant of arterial stiffening. Therefore, this genetic factor might be related to the present findings. However, flexibility levels were not associated with the 5-year progression of SBP (low: 3 ± 1, middle: 2 ± 1, high: 3 ± 1 mmHg, *P* for trend = 0.495) in the present study. Further research is necessary to determine the relationship between R577X genotype and age-related arterial stiffening. In general, there are gender differences in age-related arterial stiffening (AlGhatrif et al., 2013). We performed ANCOVA analyses by sex. Similar to previous findings (Nishiwaki et al., 2014), our results seem to indicate a stronger correlation in men. It is not clear why men show a stronger relationship between flexibility and arterial stiffness. Estrogen may has a key role in the relationship between body flexibility and arterial stiffness. However, the effect of estrogen on arterial stiffness is not consistent (Crews and Khalil, 1999; Rodriguez-Macias et al., 2002; Tatchum-Talom et al., 2002). Aortic remodeling is related to sex and women display less aortic dilation than men (Lam et al., 2010). Therefore, age-related arterial wall remodeling may be associated with our findings. Further research is necessary to determine the gender differences.

We speculate on several possible reasons for the greater progression of aortic stiffening in the low-flexibility group. First, flexibility and arterial wall stiffness at least partly interact through connective tissue metabolism (Nichols and O'Rourke, 2005). Age-related structural alterations in the arterial wall may show a consistent link with age-related alterations in body flexibility within the same individual. Second, aortic stiffness is functionally influenced by vasomotor tone (Nichols and O'Rourke, 2005). Passive muscle stretch leads to an increase in sympathetic nerve activity via the central nervous system (Yamamoto et al., 2004). Repetitive sympathoexcitation induced by habitual stretching exercises may be related to the improvements in sympathetic control of vasomotor tone (Wong and Figueroa, 2014). These improvements in sympathetic nerve control may result in a decrease in arterial stiffness (Sugawara et al., 2009). Third, it is possible that physical inactivity also contributes to this relationship. In the present study, we found that daily time spent in moderate and vigorous physical activity was longer in the high-flexibility group than in the low-flexibility group. Previous studies have indicated that moderate-to-vigorous physical activity is associated with age-related arterial stiffening (Seals et al., 2009; Gando et al., 2010). However, the present findings revealed a statistically significant inverse relationship between trunk flexibility and aortic stiffening, independent of moderate and vigorous physical activity. Fourth, PWV is also affected by other physiologic factors, such as endothelial-mediated vasodilation function and structural alterations in the arterial wall. Further research is needed to understand the association between flexibility levels and arterial function and structure.

We believe that our findings have potentially important implications for preventive and preemptive medicine. Trunk flexibility can be non-invasively, easily, quickly, and safely evaluated over all ages and in many settings. Thus, the measurement of flexibility may be useful for the screening and prevention of age-related arterial stiffening. Additional research on the mechanisms underlying the relations between flexibility and the progression of age-related arterial stiffening will help to explain these associations. If research continues to confirm causal links between flexibility and the progression of age-related arterial stiffening, it may have a positive public health impact and aid in the detection of preclinical markers of arterial stiffening. Moreover, high flexibility can be achieved through stretching or yoga, which need not be cardiorespiratory fitness-enhancing exercise (e.g., aerobic exercise), and therefore may be a practical and achievable preventive strategy in older people. Studies have indicated that acute (Yamato et al., 2016) and chronic stretching exercise (Cortez-Cooper et al., 2008; Nishiwaki et al., 2015) and yoga (Patel and North, 1975; Patil et al., 2015) significantly increased arterial compliance and reduced arterial stiffness and blood pressure. Additional research is needed to determine whether these practices may represent a new preventive and/or treatment strategy for age-related arterial stiffening. Nonetheless, we should emphasize that cardiorespiratory fitness-enhancing exercise training is an important approach for preventing aortic stiffening. In addition, the difference in 5-year change in cfPWV data between the low- and high-flexibility groups was 62 cm/s/5 years. A previous meta-analysis (Vlachopoulos et al., 2010) suggested that an increase in aortic PWV by 100 cm/s was associated with increases of 14, 15, and 15% in cardiovascular events, cardiovascular mortality, and all-cause mortality, respectively. Therefore, our findings suggest that poor flexibility exposure may be associated with future cardiovascular health.

There are several limitations to the present study. First, the sit-and-reach test used as an indicator of flexibility is a multifactorial test, which may compromise the interpretation of information. We did not evaluate the flexibility of other body regions such as the elbow, shoulder, knee, or ankle. Further studies are required to refine our understanding of the link between flexibility and arterial stiffness. Second, arterial stiffness is influenced by the phases of the menstrual cycle. We could not control for the menstrual cycle in this study. However, the number of premenopausal women was relatively low (32%), and there were no significant differences among the three groups. Therefore, we think that this factor had a small effect on aortic stiffening. Third, there were no significant differences in baseline cfPWV among the three groups (*P* = 0.577). Because the present study included subjects with a broad age range (27–66 years), our data may be less sensitive to cfPWV compared with previous reports. Our previous study suggested that trunk flexibility was correlated with arterial stiffness in older adults, but not in young adults (Yamamoto et al., 2009). Nishiwaki et al. (2014) observed a relationship between flexibility and arterial stiffness in men and elderly women. Thus, we confirmed the flexibility-arterial stiffness relationship according to age and sex. Pearson's correlation coefficients between baseline sit-and-reach and cfPWV were, in men, *r* = −0.05 (young) and −0.32 (older) and, in women, *r* = −0.02 (young) and −0.13 (older). These results seem to show greater sensitivity to cfPWV in older men. Therefore, there might be no significant differences in baseline cfPWV among the three groups. Fourth, we carried out this study as a sub-analysis of the NEXIS, which is an ongoing prospective study. Therefore, we used an observational study design. More research is needed to determine cause-and-effect relationships between flexibility and arterial stiffness.

## Conclusions

The present longitudinal study suggests that poor flexibility is associated with greater progression of age-related aortic stiffening. This association was independent of known confounders including cardiorespiratory fitness. Therefore, trunk flexibility may be an effective measure for preventing age-related aortic stiffening.

## Author contributions

YG designed the work, acquired, analyzed and interpreted the data, and wrote the first draft of manuscript. HM planned, supervised the study, and acquired the data. KY conceived the idea for the study, wrote the first draft of the manuscript, and acquired and interpreted the data. RK, HO, and NM acquired and interpreted the data. SS interpreted the data. MM designed the work, interpreted the data, wrote the first draft of the article, and supervised the study. All authors read and approved the final manuscript.

### Conflict of interest statement

The authors declare that the research was conducted in the absence of any commercial or financial relationships that could be construed as a potential conflict of interest.

## Abbreviations

ANCOVA: analysis of covariance
ANOVA: analysis of variance
BMI: body mass index
cfPWV: carotid and femoral pulse wave velocity
HbA1c: glycated hemoglobin
HDL: high-density lipoprotein
HR: heart rate
METs: metabolic equivalents
PWV: pulse wave velocity
SBP: systolic blood pressure.
